# Persistent physical symptoms reduction intervention: a system change and evaluation in secondary care (PRINCE secondary) – a CBT-based transdiagnostic approach: study protocol for a randomised controlled trial

**DOI:** 10.1186/s12888-019-2297-y

**Published:** 2019-10-22

**Authors:** Trudie Chalder, Meenal Patel, Kirsty James, Matthew Hotopf, Philipp Frank, Katie Watts, Paul McCrone, Anthony David, Mark Ashworth, Mujtaba Husain, Toby Garrood, Rona Moss-Morris, Sabine Landau

**Affiliations:** 1 Department of Psychological Medicine, Institute of Psychiatry, Psychology and Neuroscience, King’s College London, 16 De Crespigny Park, London, SE5 8AF UK; 2 Department of Biostatistics and Health Informatics, Institute of Psychiatry, Psychology and Neuroscience, King’s College London, London, UK; 3 Department of Behavioural Science and Health, University College London, London, UK; 4 Health Economics, Institute of Psychiatry, Psychology and Neuroscience, King’s College London, London, UK; 5 Division of Psychiatry, University College London, London, UK; 6 Population Health and Environmental Sciences, Faculty of Life Sciences and Medicine, King’s College London, London, UK; 7 South London and Maudsley NHS Foundation Trust, London, UK; 8 Department of Rheumatology, Guy’s and St Thomas’ NHS Foundation Trust, London, UK; 9 School of Health Psychology Section, Institute of Psychiatry, Psychology and Neuroscience, King’s College London, London, UK

**Keywords:** Medically unexplained symptoms, Cognitive behavioural therapy (CBT), Secondary care, Randomised controlled trial (RCT)

## Abstract

**Background:**

Persistent physical symptoms (PPS), also known as medically unexplained symptoms (MUS), affect approximately 50% of patients in secondary care and are often associated with disability, psychological distress and increased health care costs. Cognitive behavioural therapy (CBT) has demonstrated both short- and long-term efficacy with small to medium effect sizes for PPS, with larger treatment effects for specific PPS syndromes, including non-cardiac chest pain, irritable bowel syndrome (IBS) and chronic fatigue syndrome (CFS). Research indicates that PPS conditions share similar cognitive and behavioural responses to symptoms, such as avoidance and unhelpful beliefs. This suggests that a transdiagnostic approach may be beneficial for patients with PPS.

**Methods:**

A randomised controlled trial (RCT) will be conducted to evaluate the efficacy and cost-effectiveness of a transdiagnostic CBT-based intervention for PPS. 322 participants with PPS will be recruited from secondary care clinics. Participants stratified by clinic and disability level will be randomised to CBT plus standard medical care (SMC) versus SMC alone. The intervention consists of 8 CBT sessions delivered by a qualified therapist over a period of 20 weeks. Outcomes will be assessed at 9, 20, 40- and 52-weeks post randomisation. Efficacy will be assessed by examining the difference between arms in the primary outcome Work and Social Adjustment Scale (WSAS) at 52 weeks after randomisation. Secondary outcomes will include mood, symptom severity and clinical global impression at 9, 20, 40 and 52 weeks. Cost-effectiveness will be evaluated by combining measures of health service use, informal care, loss of working hours and financial benefits at 52 weeks.

**Discussion:**

This trial will provide a powered evaluation of the efficacy and cost-effectiveness of a transdiagnostic CBT approach versus SMC for patients with PPS. It will also provide valuable information about potential healthcare pathways for patients with PPS within the National Health Service (NHS).

**Trial registration:**

ClinicalTrials.gov NCT02426788. Registered 27 April 2015. Overall trial status: Ongoing; Recruitment status: No longer recruiting.

## Background

Medically unexplained symptoms (MUS) refer to persistent bodily symptoms cannot be adequately explained by organic pathology [1]. Although the umbrella term MUS is commonly used in health care, previous literature suggests that patients prefer the term *persistent physical symptoms (PPS)* [2]. For this reason, the patient-centred label PPS will be used throughout this paper to refer to MUS.

In secondary care, it is estimated that up to 50% of new referrals experience PPS [3]. These symptoms are often associated with profound functional impairment and psychological distress [3, 4]. Approximately 50% of patients with PPS present with co-morbid conditions, including anxiety, depression [3]. Left untreated, the prognosis of PPS patients is poor [1]. PPS are frequently seen in most medical specialties: irritable bowel syndrome (IBS) in gastroenterology, non-cardiac chest pain in cardiology, fibromyalgia (FM) in rheumatology, respiratory cough and breathlessness in respiratory medicine, and functional neurological symptoms in neurology [5]. Large amounts of healthcare and financial benefits go towards the diagnosis and treatment of PPS, with NHS costs amounting to approximately £3 billion per annum in the working population. Output losses due to sickness absence amounts to several billion [6]. Overall quality of life in people with PPS is poor with many not being able to do the things healthy people take for granted.

The management and treatment of patients with PPS is one of the most difficult tasks facing medical specialists in secondary physical healthcare services. An increasing body of evidence shows that cognitive behavioural interventions can be effective in reducing PPS severity and healthcare-related expenditures. Cognitive behavioural therapy (CBT) has been demonstrated to have both short- and long-term efficacy with small to medium effect sizes. Two meta-analyses examined the clinical effectiveness of short-term psychological therapies (including CBT, reattribution training, psychodynamic therapy, hypnosis etc.) for treating patients with PPS. The results indicated a beneficial effect of CBT for PPS [7, 8]. Improvements have also been reported for several specific PPS syndromes, including non-cardiac chest pain [9–11], IBS [12, 13], and chronic fatigue syndrome (CFS) [14]. However, more effective treatments undoubtedly need to be developed as effect sizes are at best modest. People with different PPS conditions often share similar cognitive and behavioural responses to symptoms, including fear avoidance beliefs and catastrophic misappraisal [1]. This suggests the possibility of transdiagnostic aetiological factors underlying these disorders, as well as common perpetuating processes and pathways.

**Fig. 1:**
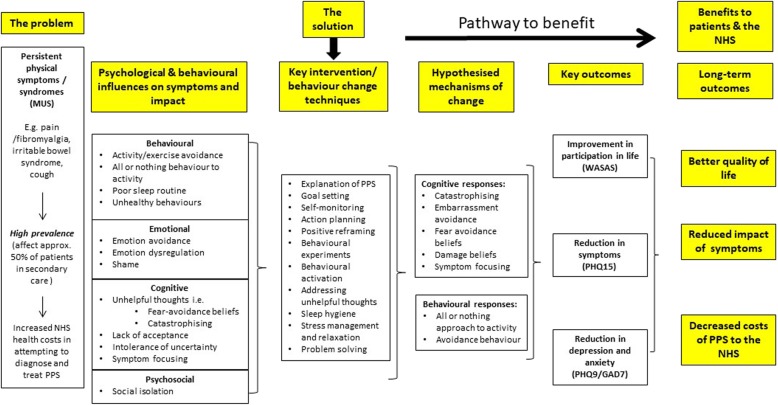
Logic Model of PRINCE Secondary illustrating the potential benefits of using a transdiagnostic approach. MUS, Medically Unexplained Symptoms; NHS, National Health Service; WSAS, Work and Social Adjustment Scale; PHQ-9, Patient Health Questionnaire – 9 item Scale; GAD-7, Generalised Anxiety Disorder – 7 item Scale; PHQ-15, Patient Health Questionnaire – 15 item Scale

There is an accumulating evidence base supporting the efficacy of transdiagnostic approaches for affective disorders. A transdiagnostic approach assumes that similar psychological processes maintain symptoms and disability across conditions. A previous systematic review and meta-analysis concluded that transdiagnostic approaches may be as effective as diagnosis-specific treatments for alleviating anxiety and may even be superior for treating depression. However these conclusions were based on a small number of studies [15]. Norton & Berra (2012) conducted a non-inferiority RCT comparing the efficacy of transdiagnostic CBT to disorder specific CBT for anxiety disorders. The results revealed treatment equivalence between transdiagnostic and disorder specific approaches, providing support for the efficacy of transdiagnostic treatments [16]. Given the large overlap between PPS syndromes, transdiagnostic interventions are needed, which can effectively target these factors to alleviate PPS, increase participation in life and reduce healthcare costs. Fig. 1 is a proposed logic model for this trial, and it illustrates the potential benefits of using a transdiagnostic approach for patients with PPS.

This paper presents a study protocol for the PRINCE (Persistent physical symptoms Reduction Intervention: a system Change and Evaluation in secondary care) Secondary trial, which aims to investigate the efficacy and cost-effectiveness of a therapist delivered transdiagnostic cognitive behavioural approach for treating patients with PPS in secondary care. Common transdiagnostic processes will be targeted with a view to bringing about a change in functional impairment and therefore will be measured alongside primary and secondary outcomes. These transdiagnostic process have been previously found to mediate change in chronic fatigue and chronic pain [17–19].

## Main research question

What is the efficacy and cost-effectiveness of a therapist delivered, transdiagnostic CBT intervention plus standard medical care (SMC) versus SMC alone for the treatment of patients with PPS in secondary medical care?

## Research objectives

### Primary objectives

1. To assess the efficacy of a therapist delivered, PPS specific (i.e. transdiagnostic) CBT intervention plus SMC for improving daily functioning in patients with PPS compared to SMC alone at 52 weeks after randomisation.

### Secondary objectives

1. To estimate whether therapist delivered transdiagnostic CBT plus SMC has a positive impact on symptom severity, mood and self-reported experience of the main symptom compared to SMC alone at 9, 20, 40 and 52 weeks post randomisation.
2. To estimate the cost-effectiveness of therapist delivered transdiagnostic CBT plus SMC versus SMC alone at 26 weeks prior-, as well as 9, 20, 40 and 52 weeks post randomisation.
3. To evaluate patients’ self-rated global measure of change in health resulting from CBT plus SMC versus SMC alone at 9, 20, 40- and 52-weeks post randomisation.
4. To assess changes in psychological distress caused by patient-defined self-rated main problems at 9, 20, 40- and 52-weeks post randomisation.
5. To assess treatment fidelity of the manual-based CBT intervention and to determine the implications for potential rollout in the National Health Service (NHS) if there is evidence of a treatment effect.
6. To investigate putative cognitive and behavioural mediators of change.

## Methods

### Trial design

A two-arm parallel group randomised controlled trial (RCT).

### Method

Three hundred and twenty-two patients with PPS will be individually randomised to CBT plus SMC versus SMC alone. Follow-up assessments will be conducted at 9, 20, 40- and 52-weeks post randomisation.

### Setting

This is a multi-centre trial. Treatment will take place at a hospital out-patient department or via telephone. Participants will be recruited from secondary care in London, UK. Clinics include rheumatology, cardiology, respiratory, neurology, gastroenterology, urology or other.

### Target population

#### Inclusion criteria

- Adults aged 18-65 years with a diagnosis of PPS. PPS is defined as persistent bodily symptoms with no clear cut obvious organic cause [1].
- Scoring ≥10 on the Work and Social Adjustment Scale (WSAS);
- Able to read and write in English;
- Willing to complete all trial visits;
- Willing and able to give written informed consent.

#### Exclusion criteria

- Active psychosis and/or factitious disorder;
- Headaches as their main and only PPS symptom (given the clinical complexity of differentiating headaches and migraine, headaches will be excluded)
- Non-epileptic seizures as their main and only PPS symptom. This is due to a large ongoing RCT evaluating a specific cognitive behavioural approach for Dissociative Seizures recruiting from some of the same clinics;
- Drug or alcohol dependence disorder;
- Use of benzodiazepines exceeding the equivalence of 10 mg diazepam per day;
- Being in receipt of or having received CBT interventions for PPS during the past year;
- Are at imminent risk of self-harm;
- Participated in PRINCE Primary study (Trial Registration Number: NCT02444520).

#### Withdrawal criteria

Participants will be withdrawn from the trial if there are any concerns regarding their consent. Participants can decide to withdraw from the study at any point of the trial, without stating a reason. The trial team will be informed if a participant decides to withdraw consent for research follow-up. Patients who discontinue their treatment but do not withdraw from the study will be followed up. Patient withdrawal forms will be used to record potential dropouts and where possible the reason for drop out.

### Planned intervention: Transdiagnostic cognitive Behavioural therapy

The intervention will consist of a maximum of 8 one-hour CBT sessions delivered by one of three qualified trial therapists over a period of 22 weeks. Ideally, CBT sessions will be delivered face-to-face every fortnight. Telephone/skype appointments will be offered in exceptional cases (e.g. for patients with mobility issues). The treatment approach will be transdiagnostic and will be flexible to accommodate the needs of the individual and specific issues associated with specific problems. Thus, it will focus on previously identified symptoms that are overlapping across patient groups included in the trial, as well as common cognitive and behavioural responses to symptoms. The treatment will be manualised to aid the standardisation of treatment delivery and facilitate a potential rollout within the NHS and other healthcare systems if found to be effective. CBT sessions will be structured according to four distinct stages: (1) engagement and rationale giving; (2) reducing avoidance by exposure techniques; (3) dealing with symptom-related cognitions and emotions; and (4) relapse prevention. Overall, the intervention aims to help patients to:

- Develop an understanding of the relationship between cognitive, emotional, physiological and behavioural aspects of their problem,
- Understand factors that may be maintaining the problem,
- Learn how to modify behavioural and cognitive responses, which may be maintaining the problem,
- Engage in avoided activities
- Address negative thoughts and illness attributions maintaining symptoms,
- Address emotional dysregulation, anxiety, low mood or low self-esteem, if present,
- Adopt a healthy sleep routine which often maintains symptoms and disrupts healthy living.
- Find ways of living with uncertainty

A therapy manual specifically designed for participants will supplement the content of CBT sessions and will act as an aid to the therapist (see Table 1 for a summary). However, the transdiagnostic approach will also be flexible to accommodate specific issues associated with specific problems. For example, participants with IBS may benefit from discussing bowel related problems.

**Table 1:** Summary of patient manual: a transdiagnostic approach for PPS

Chapter 1: Explanation of PPS	Explanation of i) PPS), ii) commonalities between PPS conditions, iii) CBT.
Chapter 2: Making sense of PPS	The impact of PPS on psychosocial functioning.
	Making a link between symptoms, behaviours and thoughts.
Chapter 3: Goal setting	Identifying goals.
	Strategies: goal setting.
	Homework: self-help materials (e.g. goal sheets).
Chapter 4: Monitoring your daily life	Rationale for keeping daily diaries.
	Homework: Keeping daily diaries.
Chapter 5: Activity scheduling	Explanation and evaluation of how PPS can reduce activity.
	Benefits of increasing activities.
	Homework: Increasing pleasurable and enjoyable activities.
Chapter 6: Overcoming barriers to change	Strategies to increase motivation.
	The impact of stress on PPS.
	The benefits of being active.
	Strategies to reduce stress levels and increase energy levels.
	Explanation and identification of “boom and bust behaviour”.
Chapter 7: Managing unhelpful thoughts and behaviours	Identifying unhelpful thoughts and behaviours.
	Self-help strategies for managing unhelpful thinking and behaviours.
	Identifying sources of social support and unhelpful relationships.
	The importance of assertiveness.
	Strategies to become more assertive.
Chapter 8: Living with uncertainty & developing acceptance	Strategies to cope with uncertainty.
	Managing discomfort with acceptance.
Chapter 9: Improving sleep	Identifying sleep problems.
	Advice on sleep management.
	Homework: Sleep management worksheets.
Chapter 10: Responding differently	Refocussing attention and distraction.
	Basic stress management.
	Relaxation techniques.
Chapter 11: Managing and coping with difficult emotions	Identifying difficult emotions.
	Coping strategies to facilitate the management of difficult emotions.
Chapter 12: Managing progress and managing setbacks	The importance of maintaining progress.
	Strategies to facilitate the routine application of relevant strategies learned in the manual/during therapy.
	Setting short- and long-term goals.
	Strategies for managing setbacks.

### Therapists

Three trained CBT therapists (clinical behavioural therapist or clinical psychologists) will provide CBT sessions. All therapists will receive training prior to delivering the CBT intervention to participants and supervision will be provided throughout the therapy. In case of resignation/parenting leave of a therapist, we will recruit a replacement therapist as quickly as possible. If a CBT session is missed due to therapist planned annual leave, the therapist will attempt to fit in the missing sessions within the five-month treatment period, but no more than one session will take place in any 1 week.

### Therapy training

All therapists will have previously been trained in CBT. A half day training will be required for the delivery of the intervention. Thereafter, weekly meetings will be set up at the start to ensure that all therapists are able to conceptualise PPS appropriately. Therapists will be informed of the trial protocol which will include how to deal with any protocol violations and confidential storage of audio-recordings. They will keep records of the therapy sessions in accordance with the guidelines of the clinical service in which they work and in accordance with professional guidelines.

### Therapy supervision

All therapists will receive group supervision every month with the CI (TC). During supervision, therapists will have the opportunity to discuss clinical issues that are problematic or challenging. The CI will remain blind to the actual identification of the patient. In addition, the supervision will check that the manual is largely being followed and ensure that the quality of therapy is sustained.

### Treatment Fidelity

All therapy sessions will be audio-recorded for treatment fidelity during the trial. A proportion of audio-recordings will be analysed by two independent clinicians once the trial has been completed. A fidelity measure will be developed, which includes overall therapeutic alliance, CBT skills and overall therapist adherence to the manual.

### Intervention adherence

The therapist will record how many CBT sessions out of 8 the participants attended, whether they were face-to-face, or telephone consultations and the duration of each session attended. At the end of therapy, the therapist will also score how well the participant adhered to therapy, as well as rate on a session-by-session basis how well the participant adhered to homework tasks.

If participants do not attend a session, the CBT therapist will contact the participant by telephone to ascertain the problem regarding attendance and will discuss options regarding how to proceed. Choices include a telephone session or a re-arranged face-to-face session, so long as the latter is within five working days. Alternatively, the session will be recorded as having not been attended. Telephone sessions will be kept to a minimum and only arranged if circumstances do not allow the patient to attend the face-to-face session.

### Standard medical care

Patients in both trial arms will receive SMC. SMC is defined as the continuation of any follow-up consultations as planned with specialised medical staff, including investigations, discussion of the PPS diagnosis, and the prescribing of any medication if required.

### Recruitment

Recruitment of patients will be from secondary care outpatient clinics or if recruitment proves difficult GP surgeries will be approached*.* Medical practitioners will inform potentially eligible patients about the trial and provide them with a trial leaflet.

### Study procedure

**Fig. 2:**
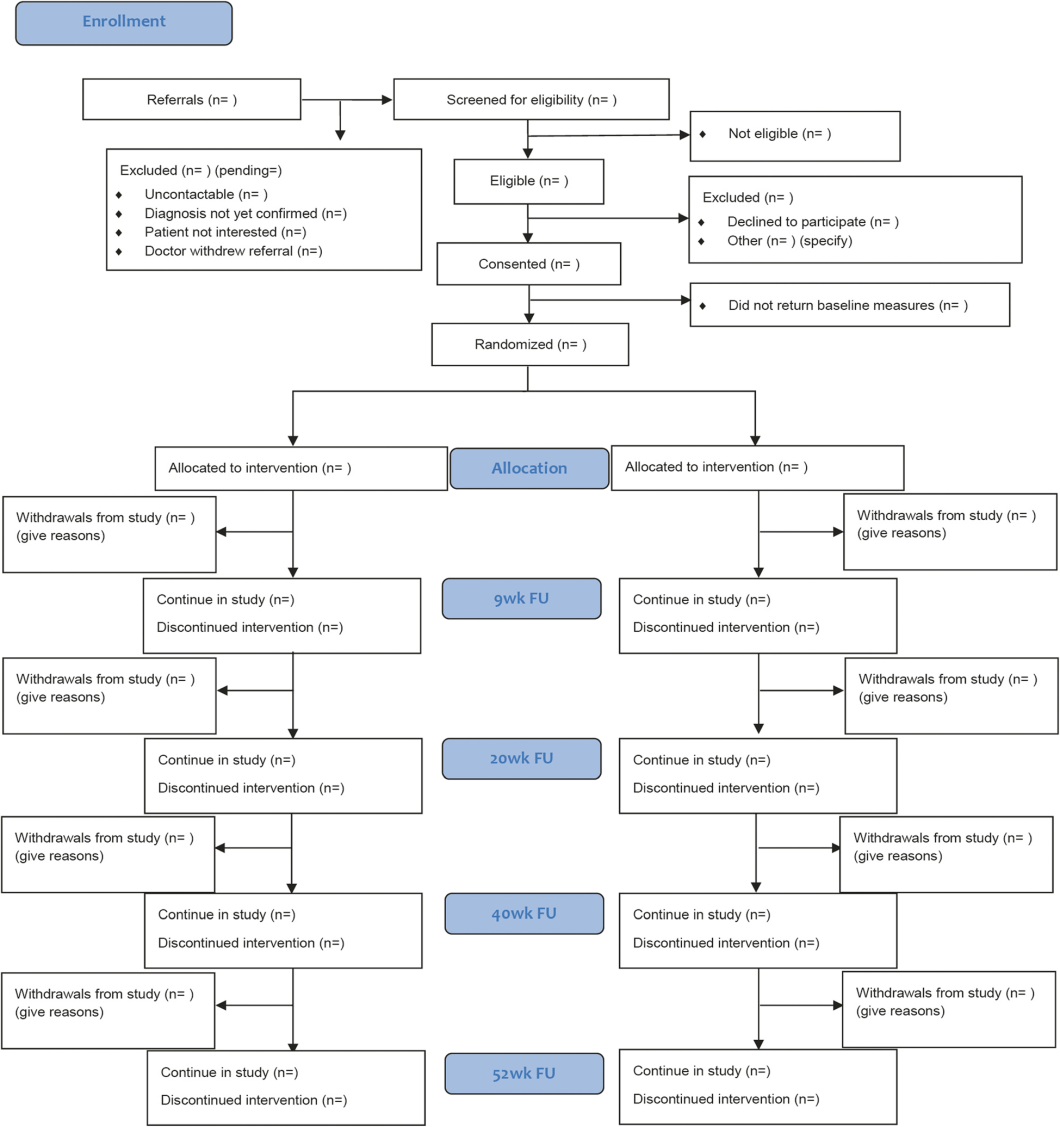
PRINCE Secondary CONSORT Diagram of Study Procedure

Interested participants will be asked to sign an agreement to be contacted by the research team. The research team will then be responsible for screening patients to check eligibility for the trial as there will be no structured clinical interview prior to inclusion. Eligible patients will then be provided with verbal and written information on the trial to read through. To formally enrol, patients will be required to complete and return a signed informed consent form. Once consent is obtained, they will be asked to complete a baseline questionnaire pack within 1 month of screening. Participants will then be randomised to one of two arms: CBT plus SMC or SMC alone. Outcomes will then be determined at 9, 20, 40 and 52-weeks after randomisation. Fig. 2 provides a CONSORT diagram, outlining the journey of all participants through the trial.

### Randomisation

Participants will be randomly allocated to one of two trial arms, using a web-based randomisation system managed by the King’s Clinical Trials Unit (KCTU). Randomisation will be at the level of the individual, using block randomisation with random block sizes, stratified by clinic (e.g., cardiology, neurology etc.) and disability level (moderately severe impairment or significant impairment) as indicated by the Work and Social Adjustment Scale.

Treatment allocation will be communicated by email to the trial manager within a period of 24 h. The trial manager will inform the participant of their treatment group by telephone/email that same day. Following allocation to the treatment group the trial manager will inform the therapist, who will contact the participant to arrange the first session.

### Proposed sample size

The sample size is based on a previous study: a RCT of rehabilitative treatments for chronic fatigue syndrome [20]. We used data from this trial as it also used the work and social adjustment as an outcome. Based on the White et al. study, we calculated a treatment effect of − 3.6 points on WSAS comparing CBT at 12 months. The within-group SD at 12 months was estimated to be 9.4 points giving a standardised effect size of Cohen’s d = 3.2/9.4 = − 0.38. The sample size calculation (Stata command sampsi) suggests that 161 patients per arm (322 in total) are needed to detect this effect size or a larger one with 90.14% power allowing for a deflation for including baseline measures in the analysis model (factor 0.84 assuming a correlation between baseline and 9-month WSAS of 0.4) and an attrition rate of 25%.

### Data management

Data management procedures and blinding will mirror the methods described in a primary care cluster randomised controlled trial [21]. This trial used the same quality assurance methods. In summary, self-report measures and therapy data will be entered onto the InferMed MACRO online database, hosted by the King Clinical Trial Unit (KCTU). This system is used for clinical trials that are managed by the Clinical trials Unit at the Institute of Psychiatry, Psychology and Neuroscience at King’s College London. It follows Good Clinical Practice and FDA 21 CFR Part 11 regulations.

Data entry and management will be undertaken by the research assistants and random checks will be undertaken by the trial manager. The Chief Investigator will be the custodian for the trial data. Patient data will be pseudo-anonymised (allocation of a unique personal identification number (PIN)) after randomisation. Data will be stored on a password-protected computer and in accordance with the General Data Protection Regulation (GDPR). Data will be archived and stored in line with requirements of the sponsor. Paper data will be stored in locked filing cabinets within a locked room and Department.

### Blinding

All data will be handled by the research team independent of the clinical team responsible for assessing and treating the patients. As this is a psychotherapy trial it is impossible to blind therapists and patients to which treatment they have been allocated to. Outcomes are all self-report measures and will be collected via post or email. Aside from the trial manger, the trial management team including the chief investigator, statisticians and the independent oversight committees will be blind to treatment allocation. If for any reason unblinding occurs the CI, TSC and DMEC will be notified.

### Data collection and follow-ups

Outcome measures will be collected at baseline (pre-randomisation) and at 9, 20, 40, and 52 weeks post-randomisation. Outcome measures will be collected during a time window and will be dependent on the follow-up time-point. Time windows will be as followed; 9 weeks – (time-window 7, 12); 20 weeks – (time-window 18, 35); 40 weeks – (time-window 38, 49); 52 weeks – (time-window 50, 63).

Participants will be asked to complete self-report measures either in writing, electronically via email or over the telephone. If participants decline to complete the full follow-up assessment but have not revoked their consent to be followed up, they will be asked to fill in only the primary outcome questionnaires. Participants who do not return their questionnaires will be contacted by the research worker via telephone/text/email, to remind them to post back their questionnaires or to invite them to complete them over the telephone.

### Measures

All measures are summarised in Table 2.

**Table 2:** Screening and data collection across the trial

	Completed by	Baseline	End of Therapy	9 weeks	20 weeks	40 weeks	52 weeks
ASSESSMENTS							
Primary Outcomes							
WSAS	P	X		X	X	X	X
Secondary Outcomes							
PHQ-15	P	X		X	X	X	X
PHQ-9	P	X		X	X	X	X
GAD-7	P	X		X	X	X	X
PPS Questionnaire	P	X		X	X	X	X
CGI-patient	P	X		X	X	X	X
CSRI	P	X		X	X	X	X
EQ-5D-5 L	P	X		X	X	X	X
Process Variables							
Therapy Process Indicators							
*Treatment Attendance and Homework Log*^b^	T						
*Treatment Adherence*^b^	T						
*Treatment Fidelity*	IC		X				
*Competence Rating*	IC		X				
Satisfaction with Treatment^a^	P				X	X	X
CGI-therapist	T		X				
PSYCHLOPS^a^	P	X		X	X	X	X
Mechanisms of Change							
*CBRQ*	P	X		X	X	X	X
*Acceptance Scale*	P	X		X	X	X	X
Baseline							
Demographic Variables	P	X					
Clinical Information	P	X					
Preferred Treatment Group	P	X					
Therapist Background Measures	T	X					
Other							
Concomitant Medications	P	X		X	X	X	X
Serious/Adverse Events	P				X	X	X

*WSAS* work and social adjustment scale, *PPS Questionnaire* persistent physical symptoms questionnaire, *PHQ-9* patient health questionnaire – 9 item scale, *GAD-7* generalised anxiety disorder – 7 item scale, *PHQ-15* patient health questionnaire – 15 item scale, *CGI* clinical global impression scale, *CBRQ* cognitive behavioural responses questionnaire, *CSRI* client service receipt inventory, *EQ-5D-5 L* EuroQol 5 Dimension 5 Level, *PSYCHLOPS* psychological outcome profiles, *P* patient, *IC* independent clinician, *T* therapist. ^a^ Assessment only completed by participants assigned to the intervention group. ^b^ completed after each therapy session

#### Baseline

1. *Demographic Variables:* A number of baseline demographic variables will be collected in order to describe the sample. This includes gender, age, ethnicity, occupational status, educational attainment, living arrangements, native language, and the clinic that the patient was recruited from (i.e. cardiology, neurology, rheumatology, respiratory, gastroenterology, urology and other)**.**
2. *Clinical information****:*** Patients will be asked to provide clinical information on their symptoms. This includes;

- ◦ Current diagnosis
- ◦ Duration of symptoms
- ◦ Comorbid medical diagnoses
- ◦ Concomitant medication
- ◦ Fibromyalgia assessment: a 4-item measure which assesses whether the participant meets the criteria for fibromyalgia.
- ◦ Health of relative/ close friend: a 4-item measure which assesses the medical history of family and close friends (i.e. heart condition, stroke, neurological condition, condition similar fibromyalgia).
- ◦ The AUDIT alcohol consumption questions (AUDIT-C): a 3-item scale with a range of scores from 0 to 12 measuring alcohol consumption [22].

3. *Preferred treatment group****:*** Patients will be asked to indicate their treatment preference post randomisation (i.e. CBT plus SMC, SMC alone, or no preference).
4. *Therapist Background Measures:* this will include gender, professional background, number of years of experience, full time/part tome status, number of PPS patients treated, highest education level.

#### Primary outcome measures

1. *Functional Impairment*: the Work and Social Adjustment Scale (WSAS) is a 5 item scale used to measure the extent to which people’s problems interfere with their ability to carry out normal activities, go to work, partake in private and social activities and impact on relationships [23]. It is a valid and reliable scale which was chosen as it is a routine outcome measure in Increasing Access to Psychological Therapies (IAPT). It has also been used in several other randomised controlled trials evaluating psychological interventions for IBS and CFS [20, 23, 24].

#### Secondary outcome measures

1. *Physical Symptoms****:*** the Patient Health Questionnaire 15 (PHQ15) will be used to measure somatic symptoms [25]. Each item is rated on a 3- point Likert scale (0 = not bothered at all; 1 = bothered a little; 2 = bothered a lot) and the total score can range from 0 to 30 where a higher score indicates higher symptom severity. The PHQ15 is a brief well-validated tool for detecting somatisation [26].
2. *Depression:* the Patient Health Questionnaire-9 (PHQ-9) will be used to monitor and measure the severity of depression in participants [27]. Each item is rated on a 4-point Likert scale (0 = not at all; 1 = several days; 2 = more than half the days; 3 = nearly every day) and the total score can range from 0 to 27 where a higher score indicates greater depressive severity. The PHQ-9 is a reliable and well-validated measure of depression severity [26].
3. *Anxiety****:*** the Generalised Anxiety Disorder – 7 (GAD − 7) questionnaire will be used to measure the severity of GAD in participants [28]. Each item is rated on a 4- point Likert scale (0 = not at all; 1 = several days; 2 = more than half the days; 3 = nearly every day) and the total score can range from 0 to 21 where a higher score indicates greater anxiety. The GAD-7 has demonstrated reliable psychometric properties in the measurement of anxiety in the general population[29].
4. *The main presenting symptom:* The Persistent Physical Symptom Questionnaire is comprised of three scales to measure (i) severity, (ii) distress and the (iii) problematic nature of the patients main presenting symptom (e.g., chest pain). Each item is scored on a 10-point scale (from 1 = not at all to 10 = extremely). Average scores from the three scales will be used to calculate an overall interference score. This measure was adapted from the Chest Pain questionnaire, which has been previously used for patients with non-cardiac chest pain [30].
5. *Global Outcome:* the adapted Clinical Global Impression (CGI patient) will be used to measure global change. It has been used in many previous trials of psychosocial treatments [31]. This is rated on a 9-point Likert scale where 1 is completely recovered and 9 is could not get any worse.
6. *Costs (Client Service Receipt Inventory):* the self-report Client Service Receipt Inventory will be used to assess health service use, informal care, lost work time and financial benefits [32].
7. *EuroQoL 5D:* the EQ-5D is a reliable and valid tool to measure health related quality of life [33]. Each dimension (mobility, self-care, usual activity, pain/discomfort and anxiety/depression) is rated on 5 levels (1 = no problems; 2 = slight problems; 3 = moderate problems; 4 = severe problems; 5 = extreme problems). The participant will also rate their own perception of their current health on a visual analogue scale ranging from 0 to 100 (0 = the best health you can imagine to 100 = the worst health you can imagine).

#### Process variables

1. *Therapy Process Indicators*
  - *Treatment attendance and homework log:* Therapist will be asked to report
    i. Patient attendance
    ii. Reasons for session cancellation if cancelled
    iii. Mode of delivery (face to face or by telephone)
    iv. Length of session
    v. Provision of handouts
    vi. Percentage of homework completed as rated by the therapist
    vii. Number of unplanned telephone calls
  - *Treatment adherence:* Treatment adherence will be rated by the CBT therapist at the end of treatment. The therapist will rate how well the participant adhered to treatment and to what extent they accepted the therapy model. This will be rated on a 4-point Likert scale.
  - *Competence rating*: *a questionnaire completed by the fidelity raters assessing the therapist competence.*
  - *Satisfaction with treatment (CBT plus SMC only):* Satisfaction of treatment will be measured at week 20. Participants will be asked to rate how satisfied they were with the CBT treatment on a 7-point Likert scale (1 Very dissatisfied; 2 Moderately dissatisfied; 3 Slightly dissatisfied; 4 *Neither; 5 Slightly satisfied; 6 Moderately satisfied; 7 Very satisfied).*
  - *Global Improvement (therapists) (CBT plus SMC only):* At the end of treatment, CBT therapists will rate how much the participant changed since the start of the study using an adapted version of the Clinical Global Impression (CGI therapist). This will be rated on a 9-point Likert scale where is 1 is completely recovered and 9 is could not get any worse.
  - *Self-Rated Patient-Defined Problem (CBT plus SMC only)*: Participants in the CBT plus SMC group only will be asked to complete the PSYCHLOPS questionnaire. The PSYCHLOPS is a well validated and reliable patient-generated measure [34], which assesses function and well-being on problems that are reported by the participant. As the problems are significant to the participant, it is highly sensitive to change and can be measured throughout the trial. Participants are asked to describe their main problem or problems and how it affects them. All responses to questions are scored.
2. *Mechanisms of Change: A separate analysis plan will be written for the mediation analysis.*
  - *The Cognitive Behavioural Responses Questionnaire (CBRQ*) is a valid and reliable tool used to assess participants cognitive and behavioural responses to their symptoms [35]. Each item is measured on a five-point Likert scale, scored from 0 (strongly disagree) to 4 (strongly agree) where a higher subscale score indicates more unhelpful cognitions and behaviours.
  - *The Acceptance scale* is a 9-item subscale measuring pain willingness, taken from the Chronic Pain and Acceptance questionnaire [36] and adapted to focus on willingness to accept symptoms. Participants will be asked to rate each item as its applied to them on a 7-point Likert scale (0 = never true to 6 = always true); where a higher score will indicate greater acceptance.

### Data collection plan: retention

Retention rates will be monitored and potentially boosted by providing participants with options regarding completion of questionnaires. These will include via post, telephone or email. Thank-you cards will be sent mid trial and end of trial.

### Statistical analysis

A statistical analysis plan (SAP) will be developed by the statisticians and agreed with the trial team before database lock. The formal statistical analyses will estimate the difference in mean outcomes between patients randomised to CBT plus SMC and SMC by intention to treat at the various post-treatment observation time points. Estimates of effect sizes together with 95% confidence intervals will be reported. It is planned that the main statistical analysis will use a linear mixed model with maximum likelihood estimation. The data will be analysed on an intention to treat basis.

The linear mixed model will contain post-treatment measures of the primary outcome (at the four follow up time points, i.e. 9, 20, 40 and 52 weeks) as the dependent variables. Fixed effects will consist of:

- Baseline measures of WSAS;
- Trial arm;
- Dummy variables for time points (9, 20, 40 or 52 weeks);
- Trial arm Χ time interaction terms;
- Dummy variables for treatment clinic (randomisation stratifier).
- Dummy variable for disability level (randomisation stratifier)
- Dummy variables for therapist

Baseline measures of the outcome variable are included as they are known predictors of the outcome and thus should help us to gain precision for effect estimates of interest.

A random effect for participant will be entered into the model to account for correlations between the four repeated measures per participant. A more complex correlation structure (e.g. random intercept and slope model) may be considered if it proved to provide a better model fit.

Standardised effect sizes will be computed to measure the effect of treatment on primary and secondary outcomes at various assessment time points. This will be done by dividing the estimated trial arm difference by the baseline standard deviation of the measure.

We will investigate empirically whether non-adherence with CBT predicts loss to follow-up, and if this were the case, we will use multiple imputation instead of linear mixed modelling to generate inferences that are valid under a realistic missing at random assumption.

We will ensure that patterns of missing data and reasons for missingness are consistent with the CONSORT diagram. Baseline characteristics will be assessed to see if they predict missingness, if so, they will be included in the analysis model. We will check if any post randomisation variables such as compliance are predictive of missingness, and if so this will inform our missing data approach.

### Economic evaluation

This analysis aims to compare the 1) service use and costs between CBT + SMC vs SMC, and 2) and assess the cost-effectiveness of CBT + SMC in relation to SMC. Two economic measures will be used to solicit data; i.e., the CSRI and the EQ-5D-5 L. The cost of services will be calculated by combining service use data with relevant unit costs [37–39]. Informal care and lost employment will both be valued using average wage rates. The use of services will be described by reporting the number of participants (%) accessing services in each group at different study points and the mean (SD) number of contacts for those using them (i.e. excluding those with zero use). Mean (SD) costs of individual services across the whole sample will also be reported and compared between the two study groups.

Health states data elicited using the EQ-5D-5 L will be combined with population utility weights to derive quality-adjusted-life-years (QALYs) used to measure health benefits in cost-utility analyses [40]. Cost-effectiveness will be assessed by combining the costs data (separately for each perspective) with the WSAS and QALYs. Incremental cost-effectiveness ratios will be computed and uncertainty around the results addressed using cost-effectiveness planes and acceptability curves.

### Trial oversight

The trial will be overseen by the Programme Management Group (PMG), as well as two independent committees, the Data Monitoring and Ethics Committee (DMEC) and the Trial Steering Committee (TSC). All committees will be responsible for ensuring that the study is conducted in accordance with the International Conference for Harmonisation of Good Clinical Practice guidelines. The DMEC will oversee the trial data, including serious adverse events and ethics and the TSC will monitor overall progress of the trial and ensure that the study protocol is being adhered to.

### Procedures for recording and reporting serious adverse events

Adverse Events (AE) will be assessed at 20, 40 and 52 weeks after randomisation. All serious adverse events (SAE) and reactions (SAR) will be reported immediately to the Chief Investigator, sponsor and DMEC. AE’s that are defined as serious will mirror the criterion described in a primary care cluster randomised controlled trial [21].

In summary, the reporting of SAE’s will be based on the following criterion; (i) death of a participant, (ii) life threatening event (iii) hospitalisation (not including elective hospitalisation for pre-existing condition) (iv) deliberate self-harm or (v) any important medical condition which may influence the participants safety. In addition, an SAE will be reported, if the participant deteriorates in that the level of disability worsens and they are unable.to carry out important daily activities for more than 4 weeks.

All serious adverse events and reactions will be reviewed, by two members of the DMEC without the presence of the CI. If deemed necessary, the scrutinisers would then be unblinded to treatment allocation so that they can then establish whether any serious adverse events were serious adverse reactions to the transdiagnostic approach.

### Stopping rules

If deemed necessary, based on either new safety information or lack of recruitment, the trial may be prematurely stopped by the Sponsor, Chief Investigator, DMEC, TSC or Research Ethics Committee (REC). If the study is discontinued, participants enrolled onto the study will be informed and subsequently data collection will stop.

### Auditing

This trial will be compliant with the research governance framework and MRC Good Clinical Practice Guideline [41]. The data will be regularly monitored and if requested, access to source data and other documents relating to the trial will be provided to the sponsor and research ethics committee for audit/REC review processes. The chief investigator will supervise a trial manager who will be situated at the Institute of Psychiatry, Psychology and Neuroscience, King’s College London. The trial manager will monitor data collection procedures, including the level of missing data. Furthermore, they will carry out source data verification checks against the paper forms. The trial manager will supervise a research worker to ensure they are fully trained in undertaking data entry/management/cleaning procedures.

The trial statisticians will be affiliated with KCTU and will be responsible for submitting reports to the DMEC and completing the trial Statistical Analysis Plan (SAP). The KCTU SOPs guidelines will be followed which outline the randomisation system, database development and statistics.

## Ethics and dissemination

### Research ethics approval

South London and Maudsley (SLaM) Hospital have agreed sponsorship. Ethical approval has been granted by the Camberwell St Giles Ethics Committee (Reference 15/LO/0058).

### Insurance/indemnity

Standard procedures for insurance of University and NHS employees and sites, and NHS patients will apply.

### Dissemination policy

The results will be presented to healthcare professionals nationally and internationally and published in peer-reviewed journals. If the intervention is more effective than the control, we plan to offer training workshops to clinical services within the NHS. We will provide lay summaries to charities and the public via websites who already disseminate information on PPS.

## Discussion

PPS are highly prevalent in both primary and secondary care, and are associated with severe physical disability, psychological distress and high health care costs [3, 4, 6]. This paper outlines the study protocol of an RCT designed to evaluate the efficacy and cost-effectiveness of a transdiagnostic cognitive behavioural intervention for adults with PPS in secondary care. The PRINCE Secondary study will be the first trial worldwide to address the efficacy and cost-effectiveness of a manual-based, transdiagnostic, approach for PPS. If it proves to be efficacious, this treatment approach could significantly improve overall functioning in patients with PPS and may lead to substantial long-term economic benefits to the NHS. Moreover, this approach could also be potentially beneficial for treating patients with other debilitating long-term conditions, including diabetes, hypertension and chronic kidney disease.

There are several limitations that need to be considered when assessing the potential impact and implications of our findings. First, we do not have an attention control so any change that occurs in the transdiagnostic CBT group cannot be attributed to the specific contents of the intervention. Second, the present trial protocol deviates from the original protocol. The intention of this study was to recruit a representative sample of therapists to deliver the intervention. However, only three therapists were recruited as opposed to eight which was outlined in the original protocol. This protocol deviation led to the trial becoming an evaluation of the efficacy of CBT delivered by these three therapists. This change in trial objective also led us to recalculate the sample size requirement with the approval of Camberwell St Giles REC.

## Data Availability

The protocol does not contain any data. The datasets generated/analysed using this protocol will be anonymised and deposited in a repository after publication. Bona-fide researchers can apply to use the data and materials but are required to clearly specify the research question a priori. No consent was provided for sharing data with third parties.

## Abbreviations

AE: Adverse event
BABCP: British Association of Behavioural and Cognitive Psychotherapies
CBRQ: Cognitive behavioural responses questionnaire
CBT: Cognitive behavioural therapy
CFS: Chronic fatigue syndrome
CGI: Clinical global impression
CI: Chief investigator
CSRI: Client service receipt inventory
DMEC: Data monitoring & ethics committee
EQ-5D: EuroQoL 5D
FM: Fibromyalgia
GAD-7: The generalised anxiety disorder (7-item version)
GDPR: General data protection regulation
GP: General practitioner
IBS: Irritable bowel syndrome
KCTU: King’s clinical trial unit
MRC: Medical research council
MUS: Medically unexplained symptoms
NHS: National Health Service
PHQ-15: Patient health questionnaire (15-item version)
PIN: Personal identification number
PMG: Programme management group
PPS: Persistent physical symptoms
PRINCE: Persistent physical symptoms reduction intervention: a system change and evaluation
RCT: Randomised controlled trial
REC: Research ethics committee
SAE: Serious adverse events
SAP: Statistical analysis plan
SAR: Serious adverse reactions
SMC: Standard medical care
SOPs: Standard operating procedures
TM: Trial manager
TSC: Trial steering committee
UKFNS: UK Functional Neurological Symptoms
WSAS: Work & social adjustment scale
