# Correction to: Persistent physical symptoms reduction intervention: a system change and evaluation in secondary care (PRINCE secondary) – a CBT-based transdiagnostic approach: study protocol for a randomised controlled trial

**DOI:** 10.1186/s12888-020-02811-w

**Published:** 2020-08-12

**Authors:** Trudie Chalder, Meenal Patel, Kirsty James, Matthew Hotopf, Philipp Frank, Katie Watts, Paul McCrone, Anthony David, Mark Ashworth, Mujtaba Husain, Toby Garrood, Rona Moss-Morris, Sabine Landau

**Affiliations:** 1 Department of Psychological Medicine, Institute of Psychiatry, Psychology and Neuroscience, King’s College London, 16 De Crespigny Park, London, SE5 8AF UK; 2 Department of Biostatistics and Health Informatics, Institute of Psychiatry, Psychology and Neuroscience, King’s College London, London, UK; 3 Department of Behavioural Science and Health, University College London, London, UK; 4 Health Economics, Institute of Psychiatry, Psychology and Neuroscience, King’s College London, London, UK; 5 Division of Psychiatry, University College London, London, UK; 6 Population Health and Environmental Sciences, Faculty of Life Sciences and Medicine, King’s College London, London, UK; 7 South London and Maudsley NHS Foundation Trust, London, UK; 8 Department of Rheumatology, Guy’s and St Thomas’ NHS Foundation Trust, London, UK; 9 School of Health Psychology Section, Institute of Psychiatry, Psychology and Neuroscience, King’s College London, London, UK

**Correction to: BMC Psychiatry 19, 307 (2019)**

**https://doi.org/10.1186/s12888-019-2297-y**

Following publication of the original article [1], the authors identified a typographical error in the proposed sample size section. The correct sentence is given below.

‘*The sample size calculation (Stata command sampsi) suggests that 161 patients per arm (322 in total) are needed to detect this effect size or a larger one with 90.14% power allowing for a deflation for including baseline measures in the analysis model (factor 0.84 assuming a correlation between baseline and* ***52 weeks*** *WSAS of 0.4) and an attrition rate of 25%’.*
